# Malignant Hyperthermia during Thoracoscopic Pulmorrhaphy in a 70-Year-Old Man

**DOI:** 10.1155/2014/250502

**Published:** 2014-05-25

**Authors:** Michihiro Sakai, Noriko Murakami, Yuji Kitamura, Shin Sato, Hiroshi Iwama, Akira Nomura

**Affiliations:** ^1^Department of Anesthesiology, Fujisawa Shonandai Hospital, 2345 Takakura, Fujisawa, Kanagawa 252-0802, Japan; ^2^Department of Anesthesiology, Kimitsu Chuo Hospital, 1010 Sakurai, Kisarazu, Chiba 292-8535, Japan; ^3^Department of Anesthesiology, Chiba University School of Medicine, 1-8-1 Inohana, Chuo-ku, Chiba 260-8677, Japan

## Abstract

Malignant hyperthermia (MH) is a rare but potentially fatal complication that may develop under general anesthesia (GA) and is rarely reported in elderly patients. We encountered a case of mild-onset MH in a 70-year-old patient who was receiving an elective thoracoscopic pulmorrhaphy and had a history of several GA procedures. Anesthesia was induced with propofol, fentanyl, and rocuronium and maintained with sevoflurane and remifentanil. His body temperature (BT) was 37.9°C after induction. During the procedure, the end-tidal CO_2_ (ETCO_2_) increased steadily to 47–50 mmHg, presumably in response to the single lung ventilation. At the end, BT was 38.1°C and ETCO_2_ was 47 mmHg under spontaneous breathing. After extubation, the patient wheezed on inspiration and expiration, and his trachea was reintubated. Sixty minutes after surgery, BT increased to 40.5°C and the arterial blood gas analysis showed severe metabolic acidosis. Based on these findings, MH was suspected and a bolus dose of dantrolene was administered. He responded to the dantrolene, and no complications or recurrence of MH was observed postoperatively. In this patient, the initial signs of MH were so subtle that making the diagnosis of MH was difficult. A high degree of suspicion is necessary to prevent a fulminant MH crisis.

## 1. Introduction

The prevalence of malignant hyperthermia (MH) during general anesthesia (GA) is reportedly 1 : 10.000–20.000 [1, 2]. It is a rare but fatal complication that may develop during surgery under GA. Recently, the prevalence of MH in Japan was verified using a nationally representative inpatient database, the Japanese Diagnosis Procedure Combination (DPC) database. From a total population of 1.2 million surgical patients under general anesthesia, 17 patients diagnosed with MH were identified (rate, 1 : 73,000), which is a prevalence rate consistent with previous data. The prevalence of MH is relatively high in patients younger than 30 years of age, relative to that observed in patients older than 30 years [3].

## 2. Case Presentation

A 70-year-old man, 167 cm tall and weighing 64 kg, was admitted to the hospital due to a left pneumothorax. He had received surgical treatment for a right pneumothorax 4 times, and general anesthesia for these procedures had been apparently unremarkable. Preoperative blood test results were within normal ranges. On physical examination, a notably heavy subcutaneous emphysema extending from his upper trunk to his neck was observed, but no dyspnea was noted. He was scheduled for a thoracoscopic pulmorrhaphy.

The preoperative vital signs were as follows: blood pressure, 127/90 (99) mmHg; heart rate, 107 beats·min^−1^; and axillary temperature, 36.9°C. Anesthesia was induced with propofol (90 mg), fentanyl (100 *μ*g), and rocuronium (70 mg). A 37 French double-lumen tracheal tube (Broncho-Cath; Tyco Healthcare, Athlone, Ireland) was used for intubation. Masseter spasm and limb rigidity did not occur during induction. GA was maintained with sevoflurane 2% administered by inhalation and remifentanil administered intravenously at a rate of 0.25–0.35 *μ*g/kg/min; muscle relaxation was achieved with rocuronium. His lungs were mechanically ventilated. After induction of anesthesia, an arterial line was inserted. The body temperature was 37.9°C 16 min after induction. The end-tidal carbon dioxide (ETCO_2_) at this point was 42 mmHg.

Surgery began uneventfully. The ETCO_2_ was 47–50 mmHg during surgery. The slight increase in ETCO_2_ was believed to be caused by ventilation of a single lung. The procedure was completed without any problem after 2.5 h. The body temperature was 38.1°C at the end of surgery. Immediately prior to extubation, ETCO_2_ was 47 mmHg under spontaneous breathing. Following extubation, he showed an agonal breathing pattern, along with inspiratory and expiratory respiratory wheezing. We were initially concerned about possible upper respiratory tract stenosis because heavy subcutaneous emphysema still remained in his neck, and therefore, we reintubated his trachea. However, examination of his subglottis respiratory tract using a bronco fiber scope did not support our initial concern. Approximately 60 min after the end of surgery, his body temperature increased from 38.1°C to 40.5°C. The ETCO_2_ at this point was 37 mmHg, despite an increase in minute volume to 15.3 L under spontaneous breathing, and the arterial blood gas analysis showed a pH of 7.27, a PaCO_2_ of 48.0 mmHg, a PaO_2_ of 277.2 mmHg, and a base excess of −5.2 mmol/L. Because of the sudden rise in temperature (more than 1.0°C in 60 min) and the presence of severe metabolic acidosis, we determined that the patient was experiencing a malignant hyperthermia crisis. Dantrolene was administered at a dose of 1 mg/kg, and after 32 minutes, his body temperature decreased to 39.2°C, and his arterial blood gas analysis revealed a marked improvement in the metabolic acidosis. Time-trend changes in body temperature and blood gas analysis are shown in Table 1. After admission to the intensive care unit, surface cooling was started, and an additional dose of dantrolene at 0.3 mg/kg was administered. Five hours after initiating treatment for malignant hyperthermia, his body temperature returned to a normal temperature of 37.9°C. Serum myoglobin and creatine kinase levels did increase to 1180.9 ng/mL and 1947 IU/L, respectively. No clinical signs of recurrence, such as body temperature increase or metabolic acidosis, were seen. His trachea was successfully extubated on the next day.

## 3. Discussion

We encountered a case of MH in an elderly man with a previous history of several general anesthetic procedures. Previous uneventful general anesthesia does not rule out development of MH [4].

According to the clinical grading scale (CGS) of Larach et al. [5], our patient's characteristics were graded as category 5 (very likely). Since the essential biochemical abnormality of MH is characterized by an increase in the release of calcium ions in skeletal muscle cells [6], according to the international view the work-up for a “definitive diagnosis” is made using calcium-induced calcium release (CICR) evaluation in the biopsied muscle fibers (Japan) [7],* in vitro* caffeine-halothane contracture test in North America (CHCT),* in vitro* contracture test in Europe, Australia and New Zealand, Brasil (IVCT) [8], and genetically by the search for MH causative mutations (the type 1 ryanodine receptor:* ryr1*, and the dihydropyridine:* cacna1s*, see http://emhg.org/). But the patient declined to have a muscle biopsy performed because the biological assay required him to travel for the test to be done (actually, the assay is done at only 2 sites in Japan) and chose not to have the genetic mutation screening because of his high age. Because potentially alternative diagnosis to MH (i.e., neuroleptic malignant syndrome, hyperthyroidism, dehydration, heat retention, and shivering) could be excluded, the MH diagnosis was made on clinical signs in our case.

MH in our patient seemed to occur immediately after anesthetic induction, indicated by the rapid change in body temperature from 36.9°C to 37.9°C in 16 min. Unfortunately, because the body temperature change was not particularly severe (38.1°C even at the end of the surgery), 4 hours elapsed before we began to suspect MH and initiate treatment. Tachycardia and rising ETCO_2_ typically precede the increased body temperature and are considered the most reliable early signs of MH, but these signs were apparently masked by the patient's preoperative physical condition and intraoperative mechanical ventilation strategy.

Our patient showed only a mild onset under mechanical ventilation during the surgery. One possible reason for this mild onset may involve the amount of skeletal muscle in the elderly [9]. Furthermore in our patient, after the trachea was extubated, his attempt to breathe spontaneously with his natural airway likely caused a critical metabolic stimulation, which itself resulted in a severe increase in body temperature. The case in our patient was clinically atypical, but this case indicates that it is important to watch closely for signs of MH not only after induction, but also during and after operation including the PACU.

We described a case of mild-onset MH, in which the initial clinical signs may be masked and the diagnosis was significantly delayed. Anesthetists need to remain vigilant for not only the subtle early signs of MH, but also an MH crisis during the whole operative period.

## Figures and Tables

**Table 1:** Body temperature and arterial blood gas analysis before and after surgery.

	16 min after induction	End of surgery	60 min after surgery	32 min after dantrolene^i^	5 h after dantrolene^i^
Body temperature (°C)	37.9	38.1	40.5	39.2	37.9
pH			7.27	7.32	7.41
PaCO_2_ (mmHg)			48.0	44.0	40.0
BE (mmol/L)			−5.2	−3.5	0.7

^i^Dantrolene (70 mg) was administered intravenously once malignant hyperthermia was suspected.
